# Machine Learning Algorithms to Predict Breast Cancer Recurrence Using Structured and Unstructured Sources from Electronic Health Records

**DOI:** 10.3390/cancers15102741

**Published:** 2023-05-13

**Authors:** Lorena González-Castro, Marcela Chávez, Patrick Duflot, Valérie Bleret, Alistair G. Martin, Marc Zobel, Jama Nateqi, Simon Lin, José J. Pazos-Arias, Guilherme Del Fiol, Martín López-Nores

**Affiliations:** 1 School of Telecommunication Engineering, University of Vigo, 36310 Vigo, Spain; 2 Department of Information System Management, Centre Hospitalier Universitaire de Liège, 4000 Liège, Belgium; 3 Senology Department, Centre Hospitalier Universitaire de Liège, 4000 Liège, Belgium; 4 Science Department, Symptoma GmbH, 1030 Vienna, Austria; 5 Department of Internal Medicine, Paracelsus Medical University, 5020 Salzburg, Austria; 6 atlanTTic Research Center, Department of Telematics Engineering, University of Vigo, 36310 Vigo, Spain; 7 Department of Biomedical Informatics, University of Utah School of Medicine, Salt Lake City, UT 84108, USA

**Keywords:** machine learning, recurrence prediction, breast cancer, patient stratification, secondary use, structured data, unstructured data

## Abstract

**Simple Summary:**

Breast cancer is a heterogeneous disease characterized by different risks of relapse, which makes it challenging to predict progression and select the most appropriate follow-up strategies. With the ever-growing adoption of Electronic Health Records, there are great opportunities to leverage the amount of data collected routinely in electronic format for secondary purposes. Machine Learning algorithms offer the ability to analyze large amounts of data and reveal insights that might otherwise go undetected. In this study, we have applied several algorithms to predict 5-year breast cancer recurrence from health data. We compared whether taking advantage of both structured and unstructured data from health records yields better prediction results than using any of the sources separately. These algorithms are valuable tools to help clinicians effectively integrate large amounts of data into their decision-making and are key to improving risk stratification and providing personalized assistance to patients.

**Abstract:**

Recurrence is a critical aspect of breast cancer (BC) that is inexorably tied to mortality. Reuse of healthcare data through Machine Learning (ML) algorithms offers great opportunities to improve the stratification of patients at risk of cancer recurrence. We hypothesized that combining features from structured and unstructured sources would provide better prediction results for 5-year cancer recurrence than either source alone. We collected and preprocessed clinical data from a cohort of BC patients, resulting in 823 valid subjects for analysis. We derived three sets of features: structured information, features from free text, and a combination of both. We evaluated the performance of five ML algorithms to predict 5-year cancer recurrence and selected the best-performing to test our hypothesis. The XGB (eXtreme Gradient Boosting) model yielded the best performance among the five evaluated algorithms, with precision = 0.900, recall = 0.907, F1-score = 0.897, and area under the receiver operating characteristic AUROC = 0.807. The best prediction results were achieved with the structured dataset, followed by the unstructured dataset, while the combined dataset achieved the poorest performance. ML algorithms for BC recurrence prediction are valuable tools to improve patient risk stratification, help with post-cancer monitoring, and plan more effective follow-up. Structured data provides the best results when fed to ML algorithms. However, an approach based on natural language processing offers comparable results while potentially requiring less mapping effort.

## 1. Introduction

Breast cancer (BC) is the most frequently diagnosed cancer in women worldwide (over 2 million new cases in 2018) and ranks second among causes of cancer-related death in women [1]. In Europe, 404,920 new cases were diagnosed and 98,755 deaths were recorded in 2018. The current trend towards individualized screening based on individual risk assessment (European study My PeBS, American study WISDOM) [2,3] has enabled early diagnosis in around 80% of cases. Although the stage at diagnosis may be the most powerful factor in determining survival and recurrence outcomes [4], BC is a complex disease, and there are many prognostic and predictive biomarkers that need to be considered to support the most appropriate targeted intervention (e.g., neoadjuvant vs. adjuvant) or combination of treatments (e.g., chemotherapy and/or hormone therapy with or without radiotherapy) in addition to surgery.

BC subtypes are highly heterogeneous and are characterized by different risks of relapse. The Luminal A subtype is associated with an excellent prognosis, with a 10-year local recurrence and distant metastases of 3.7% and 10%, respectively [5,6,7,8]. Luminal B HER2- has a higher 10-year local recurrence (5%) and distant metastases (12–20%). Local recurrence (7.5%) and distant metastases (25.6%) occurred most often in HER2+ [5,7]. Triple-negative (10 to 20% of all BC) is the most heterogeneous and aggressive subtype. It is highly metastatic within 10 years [9,10], and metastases are observed in more than 25% of these patients [5,9]. Moreover, most triple-negative recurrences occur within five years after the diagnosis [9].

BC heterogeneity makes it difficult to predict disease progression and patient outcomes, and its management will become increasingly complex in the future, owing to all the promising research in novel biomarkers and new insights that are being produced in this field. New technologies and increased scientific knowledge would enable refining patient stratifications, which would open the doors to individualizing and personalizing treatment for each patient.

The wide adoption of Electronic Health Records (EHRs) in recent years has made available a large amount of healthcare data that is collected routinely during clinical practice. These data, traditionally used for organizational and financial management, are a highly valuable source of information that could be exploited for clinical or research purposes. Machine Learning (ML) algorithms are an efficient tool for data analysis that have the potential to harness this vast amount of data to generate new insights and provide clinicians with recommendations based on real evidence, thus helping to improve care and increase patients’ quality of life.

A growing number of ML studies have been used in the analysis of healthcare data, leading to promising performance in various applications, such as cardiac arrhythmia detection [11], prediction of diabetes mellitus [12], prediction of unplanned hospital readmission [13], medical image segmentation [14], and prediction of infectious disease [15,16].

In oncology, ML-based models are gaining adoption over the conventional statistical methods used by clinicians, as they allow researchers to unveil hidden patterns in the data by providing a greater capability to account for non-linear relationships and interaction effects that are frequent in cancer data [17].

ML has been generally applied in the diagnosis and detection of cancer, for example, to identify, categorize, or distinguish tumors [18,19,20]. More recently, a growing number of ML studies have also been applied toward cancer prediction and prognosis, such as cancer risk [21], survival [22], and recurrence [23,24].

Several studies have been conducted that apply ML algorithms to predict breast cancer recurrence. For example, Lou et al. [25] compared the performance of various ML algorithms to predict recurrence within ten years after breast cancer surgery. They analyzed several predictors, including demographic characteristics, clinical characteristics, quality of care, and preoperative quality of life, and found that Artificial Neural Networks (ANN) were superior to the other forecasting models, scoring an AUROC of 97.62%. Boeri et al. [26] used two types of models, ANN and Support Vector Machines (SVM), to predict breast cancer recurrence and survival within 32 months after surgery. SVM had the best performance for loco-regional and systemic recurrence prediction, with an accuracy rate of 95.64–96.86%; however, the sensitivity was low (0.41–0.56) due to the infrequent number of positive cases in the dataset. Yang et al. [27] proposed an approach based on ensemble methods and cost-sensitive learning to manage data imbalances. By combining both methods, they achieved high sensitivity (0.947) at the cost of a significant reduction in accuracy (0.468).

Although various predictors of breast cancer recurrence risk and several types of ML algorithms have been analyzed and evaluated, this is still an open field of research. ML algorithms are highly sensitive to input data, and predictors and risk factors may vary based on different locations, lifestyles, and available data.

Generally, most of the studies using ML for cancer prognosis are limited to the analysis of structured data from the EHR. It is commonly known that curation and preprocessing of structured data are resource-heavy requirements before ML algorithms can be applied [28]. However, clinical narratives are an underexploited data source that could provide valuable complementary information for predicting clinical outcomes. For example, clinical data such as disease severity, signs and symptoms, or family history are often just recorded in the form of free text in the EHR. Some efforts have been made to integrate heterogeneous data from both structured and unstructured sources for risk prediction [29,30], leading to improved performance prediction and a reduction of errors. In relation to cancer disease, several studies have developed NLP techniques to extract cancer-related information from clinical notes [31,32,33]. However, only in a few scenarios has the information extracted been used for prognostic prediction and compared with prediction models based on structured information alone [34,35].

In this study, we compare the performance of ML algorithms to predict five-year breast cancer recurrence based on three different sets of features: (1) semi-structured data registered in the EHR; (2) features extracted from unstructured clinical reports; and (3) a combination of both. We hypothesize that by combining structured data and concepts derived from free text, we will obtain better prediction results than if we used either of the sources separately. To evaluate this, we have used data from the EHR of a cohort of breast cancer patients from the Centre Hospitalier Universitaire de Liège (CHU de Liège).

## 2. Materials and Methods

### 2.1. Experiment Design

Our approach encompasses three steps. We first performed data preprocessing and built the three datasets used to train the models, which are described in Section 3.2, Data collection and preprocessing. Second, we trained and optimized five different classification algorithms (detailed in Section 2.4 Predictive models) for each of the datasets to identify the best-performing model across the three sets of data. In the third step, we used the best-performing model to test our hypothesis that the combined dataset performs better than structured and unstructured data alone.

### 2.2. Data Collection and Preprocessing

The EHR data used in this study were extracted from the CHU de Liège in Belgium. Unstructured EHR data were de-identified using 3M™ 360 Encompass™ System anonymization tool. This means that patient names, healthcare professional names, addresses, identifiers, and phone numbers were replaced by randomly generated entities. Dates older than 20 years were also replaced with randomly generated dates in order to hide the birthdate. More recent dates were kept in order to preserve the chronology of events during the disease and follow-up periods. The health records from the hospital were mapped to the CASIDE [36] data model, based on the healthcare standard Fast Healthcare Interoperability Resources (FHIR). The initial cohort contains a total of 3839 patients who were diagnosed with breast cancer between 2010 and 2020. The number of samples was finally reduced to 823 after removing duplicates and applying some criteria to retain valid patients: the EHR contains data on the TNM staging (clinical and/or pathological), type of treatment (surgery or/and chemotherapy or/and radiotherapy), and confirmed survival of at least 5 years after diagnosis or recurrence within this period. Finally, the data appear to be highly imbalanced, with only 13% of patients showing recurrence.

We have composed three datasets: the STR dataset, based on structured and semi-structured data from EHR; the UNS dataset, based on features extracted from unstructured clinical reports; and the COMB dataset, which is a combination of the previous two.

The STR dataset was built on relevant variables for breast cancer recurrence based on a literature review and their availability in our dataset. We can see the variables used for recurrence prediction in Table 1. Then we applied several preprocessing steps, namely:

1. Data cleaning: features with more than 20% of missing values were excluded. For those accounting for less than 20% of missing values, we applied data imputation techniques such as the use of the mode (ECOG), imputation based on similar values of other variables (cTNM, pTNM), and the use of linear regression using subsets of variables as predictors (weight, height, Ki67, ER, PR, HER2).
2. Feature transformation: different transformations were applied to the extracted data for their subsequent processing by ML algorithms. Nominal features were transformed into binary class data. Dates were transformed into numerical (age and age at diagnosis) and binary (recurrence). Some features were aggregated to derive one integrated feature, for example, in the case of BMI (Body Mass Index). A more detailed process was applied to extract comorbidities. Using all extracted diagnosis codes may present challenges when training ML algorithms due to the high number of different codes and the low representativeness of each of them in our dataset. This has been solved by mapping all the diagnoses found in the list of 31 categories used in the Elixhauser Comorbidity Index [37] and counting the number of different diagnoses per category for each patient. Finally, we retained only the categories that contained 50+ instances in the dataset.
3. Scaling: we normalized all the variables to the range 0–1 prior to modeling to help with the learning process and avoid large weight values.

For the UNS dataset, we applied Symptoma’s proprietary algorithm (see Section 3.3) to extract medical concepts from narrative reports. A total of 3364 different concepts were extracted for our cohort, including diseases, symptoms, treatments, procedures, and risk factors. For each patient, the number of times each of the concepts was extracted was counted. Afterward, we applied Chi-square for feature selection and retained the 100 most relevant features for recurrence prediction. Table 2 shows the count of the selected features by concept type (the same feature can be categorized into several types). Finally, scaling was applied as in the STR dataset.

The COMB dataset contains a joint combination of the STR and the UNS dataset.

### 2.3. Automatic Information Retrieval from Unstructured Data

The extraction of concepts from the free text included in the UNS dataset has been carried out using Symptoma’s information retrieval tool, which is developed based upon Symptoma’s core technology.

Symptoma’s AI-based information retrieval algorithms ingest free text reports from EHRs and output them as collections of relevant medical features. These features are presented as standardized concepts that enable the harnessing of previously unavailable information. For the current study, due to data access constraints related to the European General Data Protection Regulation (GDPR), the data had to be stored and processed centrally on a platform with limited computational resources. Because of these computational restraints, we have chosen the bag-of-words approach, which we combined with Symptoma’s disease ontology for keyword extraction. The relationships between symptoms, signs, risk factors, and diseases that are mapped in the ontology were exploited to define the final set of features.

Across the unstructured data contained in the 3839 patients at CHU, these algorithms extracted over two million additional features, which consisted of 3364 unique concepts. On average, for each patient, an additional 528 features were extracted.

The algorithms derived these features from various unstructured inputs, including pathological studies of breast biopsy, radiotherapy treatment plans, senology outpatient visits, nuclear medicine studies, and counciliary oncological meetings. All text was supplied in French. It should be noted that no refinement training on the data set at hand has been performed to account for local idiosyncrasies (e.g., abbreviations, documentation style) or optimization for the prediction task.

### 2.4. Predictive Models

In this study, we compared the performance of five state-of-the-art classifiers in accurately predicting the probability of recurrence [38].

#### 2.4.1. Logistic Regression (LR)

Logistic Regression is a supervised learning classification algorithm used to predict the probability of a target variable. It uses a logistic function to model the dependent variable, which should be dichotomous, i.e., there could be only two possible classes. It is a common technique used when output data are binary.

#### 2.4.2. Decision Tree (DT)

Decision Tree is a Machine Learning technique that uses a set of rules to make decisions. It is used for both classification and regression, but is mostly preferred for solving classification problems. The intuition behind Decision Trees is the use of the dataset features to create yes/no questions and continually split the dataset until all data points belonging to each class are isolated.

#### 2.4.3. Gradient Boosting (GB)

Gradient Boosting is a Machine Learning technique that aggregates an ensemble of weak individual models to create a strong predictive model. Decision Trees are usually used when doing Gradient Boosting. The objective of Gradient Boosting classifiers is to minimize the loss, or the difference between the actual class value of the training example and the predicted class value. Gradient Boosting models are one of the most widely used Machine Learning algorithms today because of their effectiveness at classifying complex datasets.

#### 2.4.4. eXtreme Gradient Boosting (XGB)

XGB [39] is an optimized implementation of the GB method that provides a more regularized form of Gradient Boosting. XGB delivers high performance as compared with GB and improves model generalization capabilities by using the strengths of the second-order derivative of the loss function, L1 and L2 regularization, and parallel computing. XGB is increasingly used by the scientific community for obtaining good prediction results with relatively little effort that are comparable to or better than those provided by other more computationally expensive models.

#### 2.4.5. Deep Neural Network (DNN)

DNNs are computational learning systems inspired by the human brain and the way neurons function together to understand inputs from the human senses. DNNs are comprised of node layers, including an input layer, one or more hidden layers, and an output layer. Each node, or artificial neuron, connects to another and has an associated weight and threshold. If the output of any individual neuron is above the specified threshold, that node is activated, and data are sent to the next layer of the network. The present study used a Multi-Layer Perceptron (MLP), which is a fully connected type of feedforward DNN that is trained using the back-propagation algorithm. MLPs are able to approximate any continuous function and are one of the most widely used neural network structures, particularly the 2-layer configuration in which the input units and the output layer are interconnected with only one hidden layer.

### 2.5. Model Building and Statistical Analysis

The methodology used to build the models from each of the three datasets is the same. In order to build and evaluate the performance of the models, we first split the datasets into two randomly exclusive sets (90% for training and 10% for testing). Then, we performed hyper-parameter optimization for each algorithm and each set of data. The grid-search strategy was applied using three rounds of stratified k-fold cross-validation with k = 6 (which means that 75% and 15% of the whole dataset were used for train and validation in each pass, respectively) to optimize the models and select the best set of hyper-parameters for each algorithm and dataset. In each pass of cross-validation, the set of data used to build the model was oversampled using the Synthetic Minority Over-sampling Technique (SMOTE) [40] in order to alleviate the problem of imbalanced data. Unlike conventional oversampling techniques, which merely replicate the minority class or remove samples from the majority class, SMOTE uses the nearest neighbor algorithm to generate fresh, synthetic data to augment the minority class from the existing examples. Specifically, the minority class was resampled to equalize the number of samples in the majority class, and k = 5 was used to generate the synthetic samples.

Once the best hyper-parameters were tuned, we refitted each model using the entire training partition, to which SMOTE was again applied. We evaluated the performance of the optimized models on the 10% test holdout by comparing the models’ performance with data that had never been seen during training or optimization phases. The performance of the classifiers was assessed in terms of four metrics, which are defined as follows (where TP = True Positives, FP = False Positives, TN = True Negatives, FN = False Negatives):

- Precision: proportion of predicted positives that are actual positive cases.

PR = (TP)/(TP + FP)

- Recall: proportion of actual positives that are correctly classified.

RE = (TP)/(TP + FN)

- F1-score: harmonic mean of precision and recall.

F1 = 2 × (PR × RE)/(PR + RE)

- Area Under the Receiver Operating Characteristic (AUROC): shows how well the probabilities from the positive classes are separated from the negative ones, i.e., how adequately predictions are ranked.

In the first analysis, we averaged the scores obtained for each algorithm (LR, DT, GB, XGB, DNN) throughout the three datasets to select the best-performing one. In our second analysis, we used the best-performing ML algorithm to validate our hypothesis of whether COMB dataset provides more discriminative power than STR and UNS alone. The statistical significance of differences in performance scores between COMB and STR datasets and between COMB and UNS datasets was calculated by applying the Wilcoxon signed-rank test, since this nonparametric version of the paired Student’s *t*-test does not require a normal distribution. Metrics used for performance comparisons included AUROC as primary outcome and F1, recall, and precision as secondary outcomes. All statistical tests were two-sided, and a *p*-value less than 0.05 was considered statistically significant. 

ML algorithms and statistical analyses were implemented using Python software and Scikit-Learn [41] and Scipy [42] libraries.

## 3. Results

### 3.1. Study Characteristics

After applying the above preprocessing, a total of 823 patients were included in the final dataset, of whom 105 presented recurrence within five years after the cancer diagnosis. Descriptive statistics of the features in the STR dataset are provided in Table 3. Continuous variables are reported with the mean and standard deviation, while categorical variables are described with absolute values and percentages. In addition, the percentage of completeness of the original data (i.e., before imputation) is also displayed.

The majority of patients (99.4%) are women, and the mean age at the time of cancer diagnosis is 60.39 years. Most of the tumor sites correspond to the upper-outer quadrant of the breast (31.5%) and overlapping sites of the breast (32.4%), and the largest proportion was in grade 3 (74.2%).

Patients with 5-year recurrence have higher Ki67 values and HER2 scores and lower PR and ER levels than those without recurrence. This is in line with published material. Ki67 is a well-known marker of cell proliferation, while HER2-positive breast cancer is characterized as aggressive and has a less favorable prognosis. In addition, their number of chemotherapies entries in the STR dataset is also significantly higher (a mean of 27.70 versus 8.42), and in general, their cTNM and pTNM stages are worse.

The top five ranked features in the UNS dataset are shown in Table 4. For each concept, the mean and standard deviation of the number of times the concept was extracted for each patient are reported. The term ‘chemotherapy’ appears as one of the most relevant risk factors for prediction, as also suggested by the STR dataset. In the UNS dataset, however, it is possible to find valuable new information that is not present in the structured STR, such as symptoms. The top five ranked symptoms in the UNS dataset are shown in Table 5.

### 3.2. ML Algorithm Selection

Once the datasets are prepared, we proceed to identify the algorithm that, in general terms, offers the best classification results, which we will use in later stages to validate our hypothesis. In order to identify the best-performing ML algorithm to accurately predict the probability of five-year breast cancer recurrence, the performances obtained for each algorithm developed with the three datasets were averaged.

The averaged precision, recall, F1, and AUROC values across all three datasets are summarized in Table 6 (individual results can be seen in Table A1 in Appendix A). The results show that of the five ML algorithms evaluated, XGB achieves the best average results, outperforming the others for all the evaluation metrics (precision = 0.900, recall = 0.907, F1 = 0.897, AUROC = 0.807). It is followed by GB (precision = 0.883, recall = 0.860, F1 = 0.870, and AUROC = 0.777) and DNN (precision = 0.883, recall = 0.897, F1 = 0.887, and AUROC = 0.713). DT (precision = 0.853, recall = 0.850, F1 = 0.847, AUROC = 0.643) and LR (precision = 0.850, recall = 0.813, F1 = 0.827, AUROC = 0.640) achieve more modest results, the latter being the lowest in all metrics.

As we can see in the table, the differences of XGB with respect to the other algorithms are notable, especially in terms of AUROC, indicating that it is able to better separate between both classes. In light of these results, we can say that the XGB classifier is the best alternative for building a model for predicting the five-year recurrence of patients with breast cancer.

### 3.3. Comparison of Datasets

We used the XGB algorithm selected in the previous stage to compare the discriminative power of three sets of features to predict breast cancer recurrence within five years: (1) structured data from the EHR (STR dataset), (2) concepts extracted from clinical notes in the EHR (UNS dataset), and (3) the combination of the previous two (COMB dataset). Prediction results obtained for each dataset are presented in Table 7.

The results show that the model built from the STR dataset performs significantly better in terms of AUROC with 0.847 (95% CI 0.843–0.852) compared with the other forecasting models. Notably, the AUROC from the COMB model is the lowest of the three, with 0.778 (95% CI 0.771–0.783).

The results are similar for the other metrics (precision, recall, and F1), in which the model based on STR once again yields notably higher performance across all three measures, with 0.926 (CI 95% 0.924–0.928), 0.928 (CI 95% 0.927–0.930), and 0.919 (CI 95% 0.917–0.921), respectively. The COMB dataset again yielded the lowest performance, only surpassing UNS in F1. All differences were statistically significant.

## 4. Discussion

The occurrence of a relapse after breast cancer treatment is devastating news for patients. It is essential to optimize therapy for this group of patients to try to prevent recurrence or prolong the time until its appearance for as long as possible, but this is only feasible if it is possible to accurately identify patients who are at high risk of recurrence. Tools for relapse prediction, such as those proposed in this study, are essential to helping clinicians better tailor strategies for monitoring cancer recurrence, make personalized treatment decisions, and carry out more effective follow-up. The present study compared the predictive power of ML models trained on three different sets of features to predict a five-year recurrence. Based on our analysis, we reject the hypothesis that the combination of features from structured and unstructured data improves prediction using the individual datasets. Conversely, the results suggest that structured, tabular data gives the best predictive performance when available. Nevertheless, good performance can also be achieved with unstructured (i.e., free text) data when structured data are not available. However, combining both sets of features does not provide any advantage in predicting five-year recurrence in patients with breast cancer.

With each passing day, the amount of healthcare data available is greater and greater, and it is unreasonable to expect the physician to integrate and assimilate all of it into his decision-making effectively. The ability of ML to analyze large and diverse datasets makes it an invaluable tool when making decisions about the care of their patients, since it allows healthcare professionals to consider more evidence than they could otherwise process and remember on their own [28,43]. In this study, we hypothesized that it is possible to reuse routinely generated healthcare data using ML models to predict cancer outcomes, which could facilitate the implementation of timely pre-emptive interventions.

Among the 5 ML algorithms evaluated in this study, XGB was found to be the one that achieved the best performance on all averaged metrics across all datasets, followed by GB and DNN. As we can see, more complex algorithms such as ensembles or neural networks yield better performance. This is possibly because of their ability to better model the non-linearities of the data. It is not surprising that GB achieves similar results to XGB since they are essentially the same algorithm. However, XGB implements DART [44], a more regularized model formalization to control overfitting, which can explain its better performance. In the literature, DNN algorithms have increased in popularity in recent years and have been the algorithm of choice for many prediction tasks in healthcare lately [45,46,47], primarily due to the performance they achieve with non-traditional, non-tabular data. However, our results are consistent with Schwartz et al. [48], who compared recent works on deep learning models to XGB on a variety of tabular datasets. The study showed that for most of the datasets, XGB outperformed deep learning models and, moreover, required less tuning.

We have applied the XGB algorithm to compare the discriminative power of three sets of features, and the results have shown that the model trained with the COMB dataset yielded the lowest performance. Thus, this indicates that the combination of structured and unstructured data sources does not provide any gain in the prediction of breast cancer recurrence in our cohort. One possible explanation for this unexpected result might be that the model could have been given too many features for the limited size of our dataset. Therefore, the amount of data would not be enough to train the model while ensuring its generalization; that is, there could be a problem of high variance.

For the other two datasets, the model trained on STR outperformed the UNS model. This may indicate that the features extracted from the free text did not provide, at the bottom line, any additional signal for the prediction of recurrence in the breast cancer cohort on top of the data already available in a structured format in the EHR. However, it should be noted that the CHU de Liège EHR contains a large amount of relevant information in a semi-structured format that could be curated and incorporated into the STR dataset, which may have contributed to improved performance in the STR model. Unfortunately, this is not the case in many hospitals, whose records are not yet very well structured and may not even contain the features that have been used in this study. Additionally, extracting and mapping data into a common format is a costly process that requires manual effort and complicates the use of models based on this type of data. Thus, the NLP-based approach could be an affordable alternative since it does not require such an expensive mapping process as the STR dataset might require, while the recurrence prediction performance is comparable to the model based on structured data. In addition, the NLP-based approach also has the advantage of being potentially easier to extend for use in predicting outcomes in other types of cancer since it does not require manual adjustment, while the dataset based on structured data entails prior identification and mapping of specific tumor biomarkers for each type of cancer. 

One limitation of our analysis is the large proportion of patients that had to be discarded from the original dataset, which has resulted in a significant reduction in the data available to evaluate the algorithms. In addition to the obvious drawback of having fewer data to train the models, this may also have introduced some degree of selection bias, which could have limited the validity of the predictions. Another limitation in this study has been the constraint on computational resources, which has prevented us from applying more advanced NLP techniques. In future studies, we would like to explore techniques such as Named Entity Recognition, Relation Extraction, and Word Embeddings, which work best with deep learning models such as Bi-LSTM [49] and Transformers such as BERT [50]. With that additional syntactic and semantic information, relevant features could be extracted in a much more sophisticated way, potentially leading to improved effectiveness of the UNS dataset. Finally, the models have been trained with data from the CHU de Liège hospital only, which is not representative of a wider population. It would be of great interest to extend this study to a variety of centers and compare the performance of the models trained using data extracted from those settings. Furthermore, there are nowadays promising biomarkers that have been proposed by the scientific community, such as neutrophil-to-lymphocyte ratio (NLR) [51] or relative eosinophil count (REC) [52,53], that could provide rich information to predict the outcome of cancer patients. It would be highly interesting to incorporate these biomarkers into our predictive models in the future, once they are validated by international clinical trials.

## 5. Conclusions

This study explores the secondary use of routinely recorded EHR data to predict 5-year recurrence in breast cancer patients using ML techniques. We have derived three datasets (structured data in patient records, features extracted from clinical notes, and a combination of the previous two) from a cohort of patients from CHU de Liège to test whether providing ML models with features from structured and unstructured sources could achieve better prediction results than either source alone. We have chosen the XGB algorithm to test our hypothesis based on a comparison made between five ML algorithms.

Contrary to what we had hypothesized, the model trained on the combined dataset yielded the lowest prediction performance. The STR dataset achieved the highest performance overall, suggesting that in the data at hand, features extracted from clinical reports do not improve the predictive capacity of the data that is stored in a structured format. However, due to the low standardization of EHRs and the high cost of mapping the data used to train the ML algorithms, the NLP-based approach could be a useful and easier-to-implement alternative with fairly good performance.

ML tools such as those built in this study hold great potential to stratify patients at risk and to help professionals in decision-making and personalization of treatment, which could lead to an increase in patient survival rates. However, future research evaluating these algorithms in larger cohorts that involve multiple centers is needed to implement them in routine research and patient care.

## Figures and Tables

**Table 1:** Summary of variables used for breast cancer recurrence prediction in the STR dataset.

Feature	Possible Values	Description
Sex	nominal: male, female	Male or female
Age at diagnosis	numerical	Age of the patient at the time of diagnosis
BMI	numerical	A patient’s weight in kilograms divided by the square of his/her height in meters
ECOG	ordinal: 1, 2, 3, 4	Eastern Cooperative Oncology Group (ECOG) performance status score. Patients’ level of functioning in terms of their ability to care for themselves, daily activity, and physical ability
Comorbidities (Elixhauser categories)	nominal: Elixhauser categories	Medical condition existing simultaneously but independently with another condition in a patient
Tumor site	nominal: C501, C502, C503, [...]	Tumor body location
Grade	ordinal: 1, 2, 3, 4	The degree of differentiation of the cancer cells
TNM staging (clinical and pathological)	categorical: T1, T2, T3, T4, TX, [...]	TNM system describes the amount and spread of cancer in a patient’s body. T: tumor size N: lymph node involvement M: presence or absence of metastases
Estrogen Receptor	numerical	Percentage of cancer cells expressing estrogen receptors in the tumor tissue sample.
Progesterone Receptor	numerical	Percentage of cancer cells expressing progesterone receptors in the tumor tissue sample.
HER2	ordinal	Human Epidermal growth factor Receptor
Ki67	numerical	Antigen KI67
No. surgeries	numerical	tumorectomy, mastectomy
No. chemotherapies	numerical	Treatment of cancer by cytotoxic and/or other drugs.
No. radiotherapies	numerical	Treatment of the tumor using X-rays.

**Table 2:** Number of selected features according to concept type.

Concept Type	Number of Features
Disease	35
Symptom	53
Medicine	16
Procedure	8
Risk factor	6

**Table 3:** Patient characteristics in the STR dataset.

Feature	Total (*n* = 823)	Non-Recurrence (*n* = 718)	Recurrence (*n* = 105)	Completeness
**Sex**				100%
male	5 (0.6%)	4 (0.6%)	1 (1.0%)	
female	818 (99.4%)	714 (99.4%)	104 (99.0%)	
**Age at diagnosis**	60.39 ± 12.71	60.58 ± 12.70	59.13 ± 12.71	100%
**BMI**	25.77 ± 4.83	25.70 ± 4.90	26.28 ± 4.28	86.15%
**ECOG**				99.88%
ECOG 0	745 (91.0%)	657 (91.5%)	88 (83.8%)	
ECOG 1	65 (7.9%)	51 (7.1%)	14 (13.3%)	
ECOG 2	9 (1.1%)	7 (1.0%)	2 (1.9%)	
ECOG 3	3 (0.4%)	2 (0.3%)	1 (1.0%)	
ECOG 4	1 (0.1%)	1 (0.1%)	0 (0%)	
**Comorbidities**				100%
hypertension uncomplicated	1.01 ± 2.81	0.99 ± 2.65	1.16 ± 3.73	
chronic pulmonary disease	0.24 ± 0.94	0.22 ± 0.90	0.34 ± 1.26	
diabetes uncomplicated	0.31 ± 1.86	0.31 ± 1.84	0.41 ± 2.04	
hypothyroidism	0.49 ± 1.92	0.45 ± 1.72	0.76 ± 2.91	
metastasic cancer	3.32 ± 10.53	2.44 ± 7.27	9.36 ± 21.67	
solid tumor without metastsis	7.60 ± 9.54	6.84 ± 8.53	12.84 ± 13.63	
obesity	0.24 ± 0.83	0.22 ± 0.60	0.37 ± 1.73	
alcohol abuse	0.19 ± 1.43	0.21 ± 1.52	0.09 ± 0.37	
**Tumor site**				100%
C500	1 (0.1%)	1 (0.1%)	0 (0%)	
C501	71 (8.6%)	63 (8.8%)	8 (7.6%)	
C502	83 (10.1%)	66 (9.2%)	17 (16.2%)	
C503	48 (5.8%)	45 (6.3%)	3 (2.9%)	
C504	259 (31.5%)	231 (32.2%)	28 (26.7%)	
C505	63 (7.7%)	57 (7.9%)	6 (5.7%)	
C506	11 (1.3%)	8 (1.1%)	3 (2.9%)	
C508	267 (32.4%)	230 (%32.2)	37 (35.2%)	
C509	20 (2.4%)	17 (2.4%)	3 (2.9%)	
**Grade**				89.79%
G1	78 (9.5%)	74 (10.3%)	4 (3.8%)	
G2	133 (16.2%)	118 (16.4%)	15 (14.3%)	
G3	611 (74.2%)	525 (73.1%)	86 (81.9%)	
G4	1 (0.1%)	1 (0.1%)	0 (0%)	
**clinical TNM**				87.61%
**T**				
T1	400 (48.6%)	369 (51.4%)	31 (29.5%)	
T2	260 (31.6%)	210 (29.2%)	50 (47.6%)	
T3	30 (3.6%)	24 (3.3%)	6 (5.7%)	
T4	28 (3.4%)	16 (2.2%)	12 (11.4%)	
TX	105 (12.6%)	99 (13.8%)	6 (5.7%)	
**N**				
N0	552 (67.1%)	503 (70.1%)	49 (46.7%)	
N1	113 (13.7%)	78 (10.9%)	35 (33.3%)	
N2	11 (1.3%)	7 (1.0%)	4 (3.8%)	
N3	10 (1.2%)	6 (0.8%)	4 (3.8%)	
NX	137 (16.6%)	124 (17.3%)	13 (12.4%)	
**M**				
M0	444 (53.9%)	371 (51.7%)	73 (69.5%)	
M1	20 (2.4%)	12 (1.7%)	8 (7.6%)	
MX	359 (43.6%)	335 (46.7%)	24 (22.9%)	
**pathological TNM**				97.33%
**T**				
T0	24 (2.9%)	19 (2.6%)	5 (4.8%)	
T1	417 (50.7%)	380 (52.9%)	37 (35.2%)	
T2	232 (28.2%)	190 (26.5%)	42 (40.0%)	
T3	35 (4.3%)	22 (3.1%)	13 (12.4%)	
T4	15 (1.8%)	12 (1.7%)	3 (2.9%)	
TX	100 (12.2%)	95 (12.2%)	5 (4.8%)	
**N**				
N0	544 (66.1%)	494 (68.8%)	50 (47.6%)	
N1	153 (18.6%)	127 (17.7%)	26 (24.8%)	
N2	48 (5.8%)	32 (4.5%)	16 (15.2%)	
N3	18 (2.2%)	9 (1.3%)	9 (8.6%)	
NX	60 (7.3%)	56 (7.8%)	4 (3.8%)	
**M**				
M0	11 (1.3%)	9 (1.3%)	2 (1.9%)	
M1	13 (1.6%)	7 (1.0%)	6 (5.7%)	
MX	799 (97.1%)	702 (97.8%)	97 (92.4%)	
**ER**	79.63 ± 34.92	82.34 ± 32.51	61.13 ± 44.28	87.85%
**PR**	53.48 ± 38.53	56.46 ± 37.78	33.13 ± 37.59	87.85%
**HER2**				87.97%
0	152 (18.5%)	133 (18.5%)	19 (18.1%)	
1+	475 (57.7%)	429 (59.8%)	46 (43.8%)	
2+	128 (15.6%)	107 (14.9%)	21 (20.0)	
3+	68 (8.3%)	49 (6.8%)	19 (18.1%)	
**Ki67**	18.07 ± 19.56	16.35 ± 17.86	29.8 ± 25.8	87.97%
**No. surgeries**	1.44 ± 0.81	1.44 ± 0.79	1.45 ± 0.93	100%
**No. chemotherapies**	10.88 ± 22.51	8.42 ± 16.41	27.70 ± 42.67	100%
**No. radiotherapies**	4.24 ± 3.43	4.15 ± 3.41	4.87 ± 3.49	100%

**Table 4:** Top five selected features in the UNS dataset.

Concept	Concept Type	Total	Non-Recurrence	Recurrence
Chemotherapy	M	6.65 ± 9.13	5.95 ± 8.92	11.47 ± 9.2
Axillary Lymphadenopathy	S	1.08 ± 3.82	0.87 ± 3.52	2.5 ± 5.22
Antineoplastic Agent	M	3.16 ± 4.91	2.82 ± 4.8	5.45 ± 5.1
Carcinoma	D	8.21 ± 6.75	7.67 ± 6.61	11.87 ± 6.59
Biopsy	P	2.44 ± 4.17	2.19 ± 3.94	4.17 ± 5.21

D = Disease, S = Symptom, M = Medicine, P = Procedure, R = Risk factor.

**Table 5:** Top five selected symptoms in the UNS dataset.

Concept	Total	Non-Recurrence	Recurrence
Axillary Lymphadenopathy	1.08 ± 3.82	0.87 ± 3.52	2.5 ± 5.22
Ulcer of Nipple	0.02 ± 0.56	0 ± 0	0.15 ± 1.56
Breast Mass	0.77 ± 3.36	0.66 ± 3.22	1.58 ± 4.11
Colon Polyp	0.04 ± 0.68	0.01 ± 0.14	0.22 ± 1.87
Lymphangitis	0.08 ± 0.88	0.05 ± 0.56	0.33 ± 1.98

**Table 6:** Performance of ML algorithms averaged across the three datasets (STR, UNS, COMB).

Algorithm	Precision	Recall	F1	AUROC
LR	0.850	0.813	0.827	0.640
DT	0.853	0.850	0.847	0.643
GB	0.883	0.860	0.870	0.777
XGB	**0.900**	**0.907**	**0.897**	**0.807**
DNN	0.883	0.897	0.887	0.713

**Table 7:** Classification results of XGB algorithm in predicting five-year breast cancer recurrence using different sets of features.

Feature Set	Precision (CI 95%)	Recall (CI 95%)	F1 (CI 95%)	AUROC (CI 95%)
STR	**0.926 (0.924–0.928)**	**0.928 (0.927–0.930)**	**0.919 (0.917–0.921)**	**0.847 (0.843–0.852)**
UNS	0.894 (0.892–0.897)	0.903 (0.901–0.905)	0.882 (0.880–0.885)	0.793 (0.787–0.799)
COMB	0.891 (0.888–0.893) *†	0.891 (0.889–0.893) *†	0.889 (0.886–0.891) *†	0.778 (0.771–0.783) *†

* Statistically significantly lower than STR; † Statistically significantly lower than UNS.

## Data Availability

The datasets analyzed during the current project are not publicly available due to legal agreements made with the providing institution. Aggregated data in the form of tables are available from the corresponding author on reasonable request and subject to institutional approval.

## Appendix A

**Table A1:** Individual and average results for ML algorithms trained on the three datasets (STR, UNS, COMB).

Algorithm	Dataset	Precision	Recall	F1	AUROC
LR	STR	0.86	0.8	0.82	0.72
	UNS	0.87	0.86	0.86	0.65
	COMB	0.82	0.78	0.8	0.55
	Average	0.850	0.813	0.827	0.640
DT	STR	0.87	0.86	0.86	0.70
	UNS	0.86	0.83	0.84	0.69
	COMB	0.83	0.86	0.84	0.54
	Average	0.853	0.850	0.847	0.643
GB	STR	0.91	0.90	0.91	0.80
	UNS	0.86	0.82	0.83	0.73
	COMB	0.88	0.86	0.87	0.8
	Average	0.883	0.860	0.870	0.777
XGB	STR	0.92	0.93	0.92	0.84
	UNS	0.89	0.90	0.88	0.80
	COMB	0.89	0.89	0.89	0.78
	Average	0.900	0.907	0.897	0.807
DNN	STR	0.91	0.92	0.91	0.75
	UNS	0.89	0.90	0.89	0.82
	COMB	0.85	0.87	0.86	0.57
	Average	0.883	0.897	0.887	0.713

## References

[1] Bray F., Ferlay J., Soerjomataram I., Siegel R.L., Torre L.A., Jemal A. (2018). Global cancer statistics 2018: GLOBOCAN estimates of incidence and mortality worldwide for 36 cancers in 185 countries. CA Cancer J. Clin..

[2] Roux A., Cholerton R., Sicsic J., Moumjid N., French D.P., Giorgi Rossi P., Balleyguier C., Guindy M., Gilbert F.J., Burrion J.B. (2022). Study protocol comparing the ethical, psychological and socio-economic impact of personalised breast cancer screening to that of standard screening in the “My Personal Breast Screening” (MyPeBS) randomised clinical trial. BMC Cancer.

[3] Esserman L.J. (2017). The WISDOM Study: Breaking the deadlock in the breast cancer screening debate. NPJ Breast Cancer.

[4] Hortobagyi G.N., Stephen B.E., Armando G. (2018). New and important changes in the TNM staging system for breast cancer. Am. Soc. Clin. Oncol. Educ. Book.

[5] van Maaren M.C., de Munck L., Strobbe L.J., Sonke G.S., Westenend P.J., Smidt M.L., Poortmans P.M.P., Siesling S. (2019). Ten-year recurrence rates for breast cancer subtypes in the Netherlands: A large population-based study. Int. J. Cancer.

[6] Liu F.-F., Shi W., Done S.J., Miller N., Pintilie M., Voduc D., Nielsen T.O., Nofech-Mozes S., Chang M.C., Whelan T.J. (2015). Identification of a low-risk luminal A breast cancer cohort that may not benefit from breast radiotherapy. J. Clin. Oncol..

[7] Tsutsui S., Ohno S., Murakami S., Hachitanda Y., Oda S. (2002). Prognostic value of c-erbB2 expression in breast cancer. J. Surg. Oncol..

[8] Tobin N.P., Harrell J.C., Lövrot J., Brage S.E., Stolt M.F., Carlsson L., Einbeigi Z., Linderholm B., Loman L., Malmberg M. (2015). Molecular subtype and tumor characteristics of breast cancer metastases as assessed by gene expression significantly influence patient post-relapse survival. Ann. Oncol..

[9] Dent R., Trudeau M., Pritchard K.I., Hanna W.M., Kahn H.K., Sawka C.A., Lickley L.A., Rawlinson E., Sun P., Narod S.A. (2007). Triple-negative breast cancer: Clinical features and patterns of recurrence. Clin. Cancer Res..

[10] Boyle P. (2012). Triple-negative breast cancer: Epidemiological considerations and recommendations. Ann. Oncol..

[11] Luz E.J.d.S., Schwartz W.R., Cámara-Chávez G., Menotti D. (2016). ECG-based heartbeat classification for arrhythmia detection: A survey. Comput. Methods Programs Biomed..

[12] Zou Q., Qu K., Luo Y., Yin D., Ju Y., Tang H. (2018). Predicting diabetes mellitus with machine learning techniques. Front. Genet..

[13] Mahmoudi E., Kamdar N., Kim N., Gonzales G., Singh K., Waljee A.K. (2020). Use of electronic medical records in development and validation of risk prediction models of hospital readmission: Systematic review. BMJ.

[14] Liu X., Song L., Liu S., Zhang Y. (2021). A review of deep-learning-based medical image segmentation methods. Sustainability.

[15] Bullard J., Dust K., Funk D., Strong J.E., Alexander D., Garnett L., Boodman C., Bello A., Hedley A., Schiffman Z. (2020). Predicting infectious severe acute respiratory syndrome coronavirus 2 from diagnostic samples. Clin. Infect. Dis..

[16] Agrebi S., Anis L. (2020). Use of Artificial Intelligence in Infectious Diseases. Artificial Intelligence in Precision Health.

[17] Moncada-Torres A., van Maaren M.C., Hendriks M.P., Siesling S., Geleijnse G. (2021). Explainable machine learning can outperform Cox regression predictions and provide insights in breast cancer survival. Sci. Rep..

[18] Othman M., and Mohd A.M.B. Probabilistic neural network for brain tumor classification. Proceedings of the 2011 Second International Conference on Intelligent Systems, Modelling and Simulation.

[19] Choi Y.J., Baek J.H., Park H.S., Shim W.H., Kim T.Y., Shong Y.K., Lee J.H. (2017). A computer-aided diagnosis system using artificial intelligence for the diagnosis and characterization of thyroid nodules on ultrasound: Initial clinical assessment. Thyroid.

[20] Mambou S.J., Maresova P., Krejcar O., Selamat A., Kuca K. (2018). Breast cancer detection using infrared thermal imaging and a deep learning model. Sensors.

[21] Stark G.F., Hart G.R., Nartowt B.J., Deng J. (2019). Predicting breast cancer risk using personal health data and machine learning models. PLoS ONE.

[22] Parikh R.B., Manz C., Chivers C., Regli S.H., Braun J., Draugelis M.E., Schuchter L.M., Schulman L.N., Navathe A.S., Patel M.S. (2019). Machine learning approaches to predict 6-month mortality among patients with cancer. JAMA Netw. Open.

[23] Alabi R.O., Elmusrati M., Sawazaki-Calone I., Kowalski L.P., Haglund C., Coletta R.D., Mäkitie A.A., Salo T., Almangush A., Leivo I. (2020). Comparison of supervised machine learning classification techniques in prediction of locoregional recurrences in early oral tongue cancer. Int. J. Med. Inform..

[24] Xu Y., Ju L., Tong J., Zhou C.M., Yang J.J. (2020). Machine learning algorithms for predicting the recurrence of stage IV colorectal cancer after tumor resection. Sci. Rep..

[25] Lou S.-J., Hou M.F., Chang H.T., Chiu C.C., Lee H.H., Yeh S.C.J., Shi H.Y. (2020). Machine learning algorithms to predict recurrence within 10 years after breast cancer surgery: A prospective cohort study. Cancers.

[26] Boeri C., Chiappa C., Galli F., De Berardinis V., Bardelli L., Carcano G., Rovera F. (2020). Machine Learning techniques in breast cancer prognosis prediction: A primary evaluation. Cancer Med..

[27] Yang P.-T., Wu W.S., Wu C.C., Shih Y.N., Hsieh C.H., Hsu J.L. (2021). Breast cancer recurrence prediction with ensemble methods and cost-sensitive learning. Open Med..

[28] Ngiam K.Y., Khor W. (2019). Big data and machine learning algorithms for health-care delivery. Lancet Oncol..

[29] Chen M., Hao Y., Hwang K., Wang L., Wang L. (2017). Disease prediction by machine learning over big data from healthcare communities. IEEE Access.

[30] Zhang D., Yin C., Zeng J., Yuan X., Zhang P. (2020). Combining structured and unstructured data for predictive models: A deep learning approach. BMC Med. Inform. Decis. Mak..

[31] Zeng Z., Espino S., Roy A., Li X., Khan S.A., Clare S.E., Jiang X., Neapolitan R., Luo Y. (2018). Using natural language processing and machine learning to identify breast cancer local recurrence. BMC Bioinform..

[32] Karimi Y.H., Blayney D.W., Kurian A.W., Shen J., Yamashita R., Rubin D., Banerjee I. (2021). Development and use of natural language processing for identification of distant cancer recurrence and sites of distant recurrence using unstructured electronic health record data. JCO Clin. Cancer Inform..

[33] Datta S., Bernstam E.V., Roberts K. (2019). A frame semantic overview of NLP-based information extraction for cancer-related EHR notes. J. Biomed. Inform..

[34] Barber E.L., Garg R., Persenaire C., Simon M. (2021). Natural language processing with machine learning to predict outcomes after ovarian cancer surgery. Gynecol. Oncol..

[35] Ribelles N., Jerez J.M., Rodriguez-Brazzarola P., Jimenez B., Diaz-Redondo T., Mesa H., Marquez A., Sanchez-Muñoz A., Pajares B., Carabantes F. (2021). Machine learning and natural language processing (NLP) approach to predict early progression to first-line treatment in real-world hormone receptor-positive (HR+)/HER2-negative advanced breast cancer patients. Eur. J. Cancer.

[36] González-Castro L., Cal-González V.M., Del Fiol G., López-Nores M. (2021). CASIDE: A data model for interoperable cancer survivorship information based on FHIR. J. Biomed. Inform..

[37] Quan H., Sundararajan V., Halfon P., Fong A., Burnand B., Luthi J.C., Saunders L.D., Beck C.A., Feasby T.E., Ghali W.A. (2005). Coding algorithms for defining comorbidities in ICD-9-CM and ICD-10 administrative data. Med. Care.

[38] Bonaccorso G. (2017). Machine Learning Algorithms.

[39] Chen T., Guestrin C. Xgboost: A scalable tree boosting system. Proceedings of the 22nd ACM SIGKDD International Conference on Knowledge Discovery and Data Mining.

[40] Chawla N.V., Bowyer K.W., Hall L.O., Kegelmeyer W.P. (2002). SMOTE: Synthetic minority over-sampling technique. J. Artif. Intell. Res..

[41] Pedregosa F., Varoquaux G., Gramfort A., Michel V., Thirion B., Grisel O., Blondel M., Prettenhofer P., Weiss R., Dubourg V. (2011). Scikit-learn: Machine learning in Python. J. Mach. Learn. Res..

[42] Virtanen P., Gommers R., Oliphant T.E., Haberland M., Reddy T., Cournapeau D., Burovski E., Peterson P., Weckesser W., Bright J. (2020). SciPy 1.0: Fundamental algorithms for scientific computing in Python. Nat. Methods.

[43] Kantarjian H., Yu P.P. (2015). Artificial intelligence, big data, and cancer. JAMA Oncol..

[44] Vinayak R.K., Gilad-Bachrach R. Dart: Dropouts meet multiple additive regression trees. Proceedings of the Artificial Intelligence and Statistics, PMLR.

[45] Tomašev N., Harris N., Baur S., Mottram A., Glorot X., Rae J.W., Zielinski M., Askham H., Saraiva A., Magliulo V. (2021). Use of deep learning to develop continuous-risk models for adverse event prediction from electronic health records. Nat. Protoc..

[46] Gupta M., Phan T.L.T., Bunnell H.T., Beheshti R. (2022). Obesity Prediction with EHR Data: A deep learning approach with interpretable elements. ACM Trans. Comput. Healthc. (HEALTH).

[47] Pham T., Tran T., Phung D., Venkatesh S. (2017). Predicting healthcare trajectories from medical records: A deep learning approach. J. Biomed. Inform..

[48] Shwartz-Ziv R., Armon A. (2022). Tabular data: Deep learning is not all you need. Information Fusion.

[49] Schuster M., and Paliwal K.K. (1997). Bidirectional recurrent neural networks. IEEE Trans. Signal Process..

[50] Devlin J., Chang M.W., Lee K., Toutanova K. (2018). Bert: Pre-training of deep bidirectional transformers for language understanding. arXiv.

[51] Gianni C., Palleschi M., Schepisi G., Casadei C., Bleve S., Merloni F. (2022). Circulating inflammatory cells in patients with metastatic breast cancer: Implications for treatment. Front. Oncol..

[52] Onesti C.E., Josse C., Boulet D., Thiry J., Beaumecker B., Bours V., Jerusalem G. (2020). Blood eosinophilic relative count is prognostic for breast cancer and associated with the presence of tumor at diagnosis and at time of relapse. Oncoimmunology.

[53] Onesti C.E., Josse C., Poncin A., Frères P., Poulet C., Bours V., Jerusalem G. (2018). Predictive and prognostic role of peripheral blood eosinophil count in triple-negative and hormone receptor-negative/HER2-positive breast cancer patients undergoing neoadjuvant treatment. Oncotarget.

